# Cloning of the Soybean *GmNHL1* Gene and Functional Analysis under Salt Stress

**DOI:** 10.3390/plants12223869

**Published:** 2023-11-16

**Authors:** Lu Liu, Jiabao Wang, Qi Zhang, Tingting Sun, Piwu Wang

**Affiliations:** 1 College of Life Sciences, Jilin Agricultural University, Changchun 130118, China; 2 The Center of Biotechnology, Jilin Agricultural University, Changchun 130118, China

**Keywords:** salt stress, *GmNHL1* gene, reactive oxygen species, physiological and biochemical indicators, microscopic structure

## Abstract

When encountered in the soybean seedling stage, salt stress has serious impacts on plant growth and development. This study explores the role of the soybean *NDR1/HIN1-like* family gene *GmNHL1* under salt stress. First, the *GmNHL1* gene was successfully cloned, and bioinformatic analysis revealed multiple cis-acting elements which are related to adversity stress and involved in the oxidative response in the promoter region. Sub-cellular localization analysis indicated that the protein expressed by *GmNHL1* was localized on the cell membrane. An over-expression vector of the target gene and a CRISPR/Cas9 gene-editing vector were constructed, and the recipient soybean variety Jinong 74 was genetically transformed using the *Agrobacterium tumefaciens*-mediated method. By analyzing the performance of the different plants under salt stress, the results showed that *GmNHL1* was over-expressed in the T2 generation. The germination potential, germination rate, germination index, and vitality index of the strain were significantly higher than those of the recipient control JN74. Under salt stress conditions, the root microanatomical structure of the *GmNHL1* over-expressing material remained relatively intact, and its growth was better than that of the recipient control JN74. Measurement of physiological and biochemical indicators demonstrated that, compared with the receptor control JN74, the malondialdehyde and O_2_^−^ contents of the *GmNHL1* over-expressing material were significantly reduced, while the antioxidant enzyme activity, proline content, and chlorophyll content significantly increased; however, the results for *GmNHL1* gene-edited materials were the opposite. In summary, over-expression of *GmNHL1* can improve the salt tolerance of plants and maintain the integrity of the root anatomical structure, thereby more effectively and rapidly reducing the accumulation of malondialdehyde and O_2_^−^ content and increasing antioxidant enzyme activity. This reduces cell membrane damage, thereby improving the salt tolerance of soybean plants. These results help to better understand the mechanism of salt tolerance in soybean plants, laying a theoretical foundation for breeding new stress-resistant varieties of soybean.

## 1. Introduction

Soybean (*Glycine max* (Linn.) Merr.) originates from China and is an essential oil crop. Salt stress is one of the main abiotic factors affecting plant growth, development, and yield [1]. In some areas of northern China, the soil salinization is severe, widely distributed, and presents an annually increasing trend. When crops are grown on saline–alkali soil, their yield and quality will vary [2]. Planting salt-tolerant plants is an efficient and environmentally friendly soil improvement method [3]. Soybeans have a short growth cycle, and planting them on land with high salinity can seriously impact their yield. Therefore, cultivating soybean varieties with strong salt tolerance can effectively increase their yield and resistance to adverse environments [4,5].

Under salt stress, an increase in soil salt solution concentration will cause the soil osmotic potential to increase, making it difficult for plant root cells to absorb water, thus resulting in physiological drought [6]. The seedling stage is a critical period in the life of soybeans. Germination is the growth period when soybeans first face salt stress. The germination and seedling stages of soybeans are more sensitive to salt stress than other periods. Therefore, the salt tolerance ability of soybeans in the germination stage is the key to growing them in saline–alkali soils, being an essential characteristic necessary for growth and development [7]. Plants can maintain increased stress resistance by increasing osmoregulation, antioxidant capacity, and dehydration tolerance [8,9]. Among them, maintaining the content of cellular reactive oxygen species (ROS) is an essential manifestation of a plant’s ability to withstand adverse stress. An increase in ROS production under stress conditions will cause cell damage [10]. The enzymatic reactive oxygen species scavenging system mainly includes peroxidase (POD), catalase (CAT), superoxide dismutase (SOD), and so on [11]. Among them, superoxide dismutase defends against reactive oxygen species inside and outside cells and can remove excess O_2_^−^ in cells [12]. The antioxidant function of POD is mainly reflected in the early stage of salt stress, as it can remove excess hydrogen peroxide (H_2_O_2_) [13]. Under salt stress, soybeans will produce an osmotic physiological response mechanism, accumulate osmotic regulatory substances, and seek to maintain the normal physiological functions of cells [14]. Substances such as inorganic ions and free proline are essential regulatory substances. Nguyen et al. have observed that over-expression of the *GmDREB6* gene allows for a response to salt stress by regulating *GmP5CS* transcription levels and Pro accumulation [15]. Jin et al. have compared the root fresh weight, primary root length, peroxidase, and superoxide dismutase activities of over-expressed *GsPRX9* plants and control plants, and found that over-expression of the *GsPRX9* gene enhanced the salt tolerance in soybeans [16]. These research results indicate that physiological activity indicators and osmotic adjustment of substance contents can be used as indirect indicators to judge the stress resistance of soybeans.

The NDR1/HIN1-like (*NHL*) gene family plays an important role in various plant defense responses [17], growth and development [18], and resistance to abiotic stresses [19]. This gene family has been shown to play an important role in stress regulation in different species. According to SMART (Simple Modular Architecture Research Tool) predictions, most *NHL* gene structures contain a conserved late embryonic development-rich domain (LEA). LEA proteins are a group of vital proteins in plants that serve crucial roles during adverse stress conditions. They accumulate in the later stages of germination to resist dehydration [20]. Therefore, the *NHL* gene containing the conserved domain of LEA may also play an important role when seeds are under adverse stress, helping to maintain normal seed germination and early growth. In 2017, Song et al. showed that Arabidopsis *NHL6* is highly expressed in ungerminated seeds, and its expression is strong under the induction of ABA and various abiotic stress signals. It was found that over-expression of *NHL6* in *Arabidopsis thaliana* increased the sensitivity of positive plants to ABA, salt, and drought [21]. In 2018, Liu et al. analyzed the function of the *CaNHL4* gene under abiotic stress. *CaNHL4* affects the production of ROS and the expression of genes related to the SA and JA pathways. Their results indicated that *CaNHL4* may be involved in activating the SA and JA signaling pathways and the production of ROS in pepper [22]. The functions of *NHL* family genes in different plants under abiotic stresses have been verified; however, a functional study of the soybean *NHL1* gene (*GmNHL1*) under salt stress conditions has not yet been reported. In summary, in this study, the target gene *GmNHL1* of the soybean *NHL* family was cloned, and sub-cellular localization was performed to clarify its protein expression location. By constructing plant over-expression vectors and CRISPR/Cas9 gene editing vectors for genetic transformation, it is found that over-expression of *GmNHL1* improved the antioxidant enzyme activity of transgenic soybeans and slowed down the accumulation of ROS, thus improving the tolerance to salt stress. This experiment may serve as a theoretical foundation for cultivating new stress-resistant varieties of soybean.

## 2. Results

### 2.1. Bioinformatics Analysis of GmNHL1 Gene

For this study, we cloned the NDR1/HIN1-like gene family gene *GmNHL1* from soybean JN74. The CDS of this gene has a total length of 624 bp, encoding a polypeptide containing 307 amino acid residues and a molecular weight of 23.92 KD. Based on the amino acid sequence of *GmNHL1*, a phylogenetic tree of plant genes was constructed, and the results indicated that it belongs to the same branch as rice, with the closest genetic relationship (Figure S1A). Multiple sequence analysis and alignment analysis showed that *GmNHL1* has high homology with genes of the same family in maize and rice (Figure S1D), and both contain the LEA-conserved binding domain related to function (Figure S1C). The upstream promoter regulates the expression level of the gene, and the upstream promoter region of the *GmNHL1* gene contains multiple elements related to stress, jasmonic acid, ABA, and light responses (Figure S1B).

### 2.2. Cloning of Target Genes

**Figure 1:**
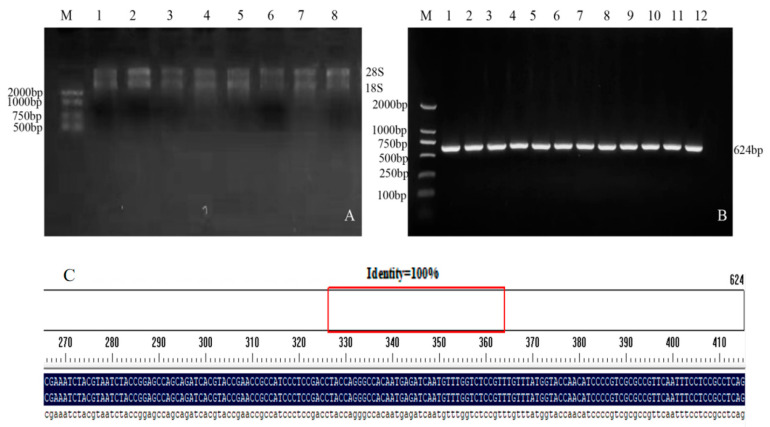
Extraction of total RNA from soybean leaves and cloning of target genes. (**A**) Total RNA extraction from soybean leaves at V2 stage; (**B**) Cloning of target gene; and (**C**) Sequencing comparison results.

In the PCR electrophoresis results, the extracted RNA showed two bands (Figure 1A). PCR amplification was performed using the cDNA of soybean Jinong 74 as a template, and a target fragment with a band size of 624 bp was obtained (Figure 1B). The sequencing results were compared with the target gene sequence using the DNAMANN software (V6.0) and the consistency was 100% (Figure 1C), proving that the *GmNHL1* gene had been successfully cloned.

### 2.3. Sub-Cellular Localization

**Figure 2:**
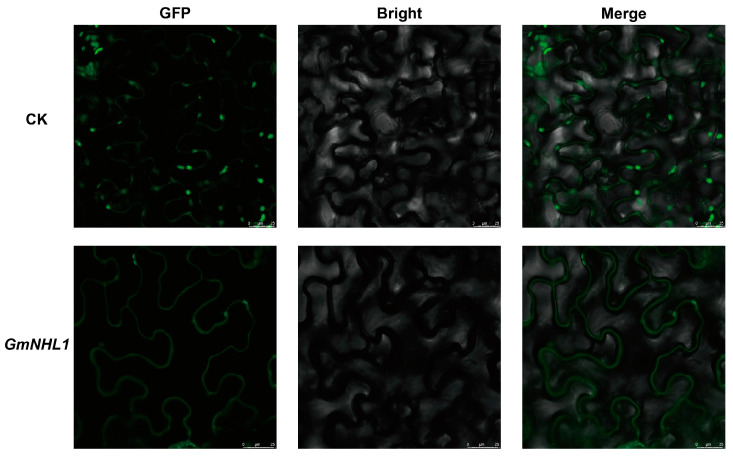
Sub-cellular mapping results of *GmNHL1* gene. Note: CK, pCAMBIA1302-GFP; *GmNHL1*, pCAMBIA1302-GmNHL1-GFP; GFP, green excitation light state; bright, bright-field; merge, superposition state.

Understanding the sub-cellular localization of gene expression proteins is of great significance for the functional analysis of genes. To determine the sub-cellular localization of *GmNHL1*, transient expression analysis of pCAMBIA1302-GFP and pCAMBIA1302-GmNHL1-GFP was performed in the leaves of Ben’s tobacco. As shown in Figure 2, the green fluorescence signal of unfused GFP in the tobacco was distributed in the nucleus and cytoplasm. In contrast, the green fluorescence signal of GmNHL1-GFP was only distributed in the cell membrane. Thus, it was confirmed that *GmNHL1* localized on the cell membrane.

### 2.4. Analysis of Gene Expression Patterns under Different Stress Conditions

**Figure 3:**
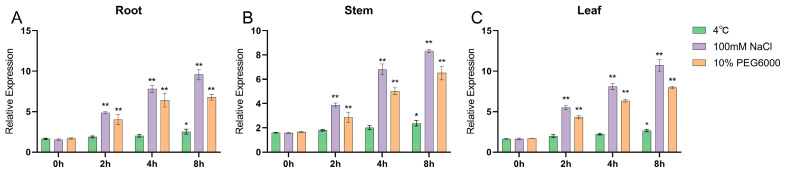
Expression of *GmNHL1* in roots, leaves, and stem of soybean at different times under different stresses. (**A**) Root; (**B**) Stem; (**C**) Leaf. * *p* < 0.05; ** *p* < 0.01.

qRT-PCR was conducted to verify the expression of the *GmNHL1* gene in soybean under different stress treatments. The experimental results indicated that the *GmNHL1* gene could be induced to express under drought, low temperature, and salt stresses, and its expression level was the highest at 8 h of stress. The Gm*NHL1* gene significantly responded to salt stress, under which its expression was upregulated (Figure 3).

### 2.5. Genetic Transformation and Identification of Positive Plants

**Figure 4:**
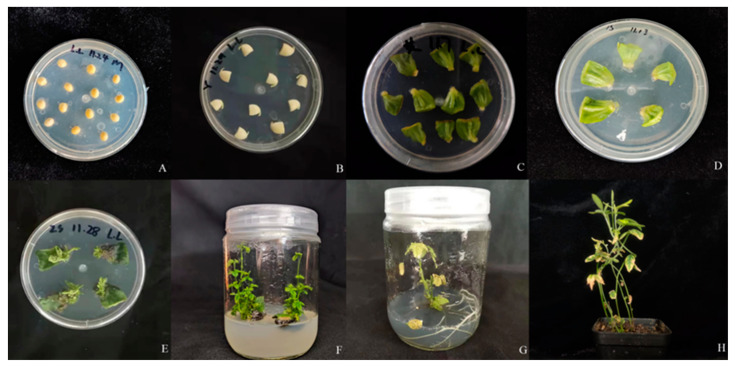
The process of soybean cotyledon node transformation using *Agrobacterium tumefaciens.* (**A**) Initiation of germination culture; (**B**) Pre-cultivation phase; (**C**) Co-cultivation stage; (**D**) Initial screening phase; (**E**) Secondary screening phase; (**F**) Elongation stage; (**G**) Rooting phase; and (**H**) Transplantation.

The genetic transformation of soybean mediated by *Agrobacterium tumefaciens* mainly consisted of eight steps: germination, pre-culture, infection, elongation, rooting, and transplanting (Figure 4).

**Figure 5:**
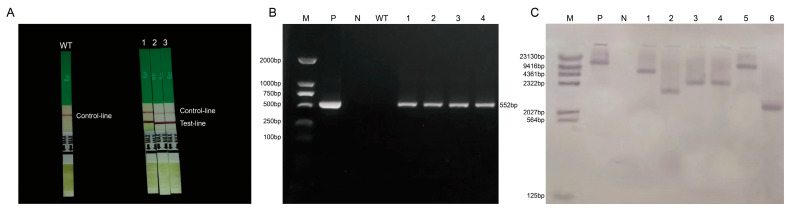
Detection of positive plants. (**A**) Bar test strip detection of positive plants in T1 generation; (**B**) PCR detection of positive plants in T2 generation; (**C**) Southern blot hybridization detection of positive plants in T2 generation. Note: M, Southern DNA marker; P, positive control; N, water; WT, untransformed plants; 1–6, transformed plants.

The T1 generation plants obtained through genetic transformation were detected using a Bar strip (Figure 5A), and PCR detected the T2 generation positive plants with the specific marker gene Bar (Figure 5B). A total of 14 positive plants were screened, and some of the test results were as follows. Southern blot hybridization was performed on T2 over-expressed transformation plants that were preliminarily positive (Figure 5C), and the test results indicated that the *GmNHL1* gene was mainly integrated into the genome of T2 generation transformation plants through single- and multi-copy methods, where the integration sites differed.

### 2.6. Fluorescence Quantitative PCR Detection of T2 Generation Positive Plants

**Figure 6:**
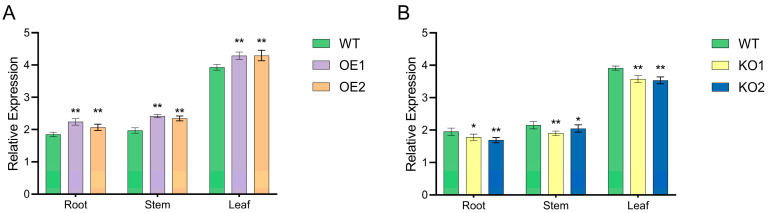
*GmNHL1* gene expression in T2 generation positive plants. (**A**) Over-expressing materials; (**B**) gene-edited materials. * *p* < 0.05; ** *p* < 0.01. Note: WT, wild-type receptor soybean variety JN74; OE1 and OE2, over-expressing soybean materials; KO1 and KO2, gene-edited soybean materials.

The qRT-PCR was conducted to detect the expression levels of target genes in the root, stem, and leaves of T2-positive plants and receptor control plants. The results showed that T2 over-expressing materials showed an increasing trend (Figure 6A); however, gene-edited materials showed a downward trend (Figure 6B). In combination with expression level detection and molecular identification, we selected OE1 and OE2 over-expressing materials with single copy numbers of target gene and significantly increased expression level, and KO1 and KO2 gene edited materials with preliminary positive identification and decreased expression level for follow-up tests.

### 2.7. Identification of Salt Tolerance in Transgenic GmNHL1 Soybean during Germination

**Figure 7:**
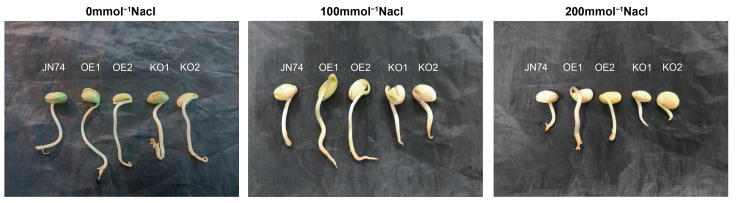
Phenotype analysis regarding germination of JN74 and *GmNHL1* transgenic soybean under different concentrations of NaCl.

As seen from Table 1, the germination potential, germination rate, and germination index in soybean seeds decreased significantly with increased NaCl concentration. Under the control condition, the germination potential and germination rate for the seeds of each strain were the highest. The difference was not statistically significant, indicating that all seeds germinated well under normal conditions (Figure 7). With increased NaCl concentration, the germination state of OE1 and OE2 materials was significantly better than that of the control, while the germination state of KO1 and KO2 materials was significantly weaker than that of the control. After treatment with 100 mmol/L NaCl, the relative germination potential of OE1 and OE2 was significantly higher than that of the control, while that of KO1 and KO2 was significantly lower than that of the control. Compared with the control treatment, the relative germination rates of JN74 and OE2 presented no significant change, while that of OE1, KO1, and KO2 decreased slightly. The germination index and vitality index of OE1 and OE2 were significantly higher than that of the control, but KO1 and KO2 were significantly lower than that of the control. Under 200 mmol/L NaCl, the germination potential, germination rate, germination index, and vitality index of OE1 and OE2 were significantly higher than those of the control, while those of KO1 and KO2 were significantly lower than the control. The results indicated that over-expression of the *GmNHL1* gene significantly improved the germination potential, germination rate, and germination index of soybean seeds under salt stress.

### 2.8. Comparative Examination of Various Soybean Strains’ Responses to Distinct Salt Stress Conditions

**Figure 8:**
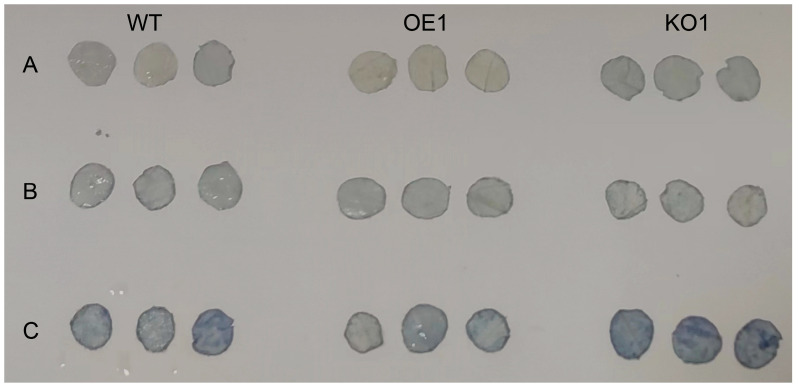
Histochemical localization of superoxide anions in the leaves of three soybean varieties at the V2 stage after 15 days of salt stress. The dark blue precipitate shows the reaction of NBT with superoxide anions to produce insoluble formic acid. (**A**) Under normal circumstances; (**B**) 100 mmol^−1^ NaCl treatment; and (**C**) 200 mmol^−1^ NaCl treatment.

**Figure 9:**
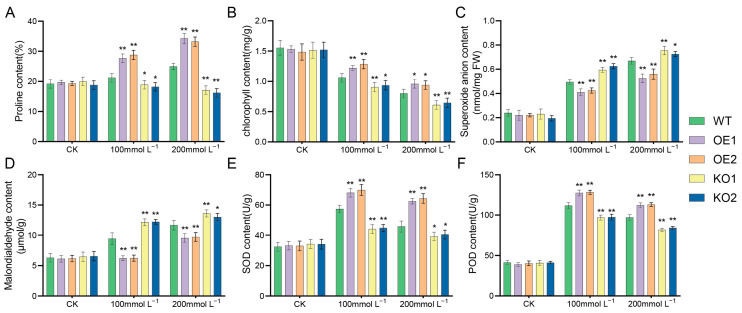
Determination of physiological and biochemical indices of different materials under salt stress. (**A**) Proline content; (**B**) ChlorophyII content; (**C**) Superoxide anion content; (**D**) Malondialdehyde content; (**E**) SOD content; (**F**) POD content. * *p* < 0.05; ** *p* < 0.01.

Plants produce more ROS under stress and increase enzymatic and non-enzymatic clearance system activities to eliminate excess ROS in the body. First, NBT staining was conducted to observe the color depth of leaves and identify the change in ROS content in leaves. The results (see Figure 8) demonstrated that, with increasing exposure to stress, the color of the gene-edited lines became significantly darker than that of the recipient lines, indicating that the ROS content in the leaves of the gene-edited lines had increased. The O_2_^−^ content was then measured; the results are shown in Figure 9C. Under normal conditions, there was no significant difference between the two. After salt stress treatment, the O_2_^−^ content of the over-expression strain plants was significantly lower, compared with the recipient plants, indicating that the reactive oxygen species content in the over-expression strain was significantly lower than that in the recipient plants; thus, the salt tolerance of the soybean plants was improved.

Plant physiological and biochemical indices change in response to salt stress. As such, the degree of salt stress in soybeans can be judged by analyzing the trends of various indices. The test results showed no significant differences in malondialdehyde content, superoxide anion content, chlorophyll content, oxide dismutase (SOD) activity, superoxide dismutase (POD) activity, and proline content among soybean plants of different strains under normal conditions (Figure 9). When treated with 100 mmol/L NaCl, there were significant differences in the various physiological indices among the materials. In addition, after salt stress, the malondialdehyde and superoxide anion contents in gene-edited materials were consistently higher than those in over-expressing materials, while the chlorophyll content, proline content, POD activity, and SOD activity in gene-edited materials were lower than in over-expressing materials. With increasing salt concentration, the change trend of each index gradually increased, showing a significant difference as a whole. The results indicated that expression of the *GmNHL1* gene can respond to salt stress and affect the salt tolerance of plants by regulating changes in physiological and biochemical indices in the plants.

### 2.9. Effects of Salt Stress at Seedling Stage (V1 Stage) on Soybean Phenotype and Microstructure

**Figure 10:**
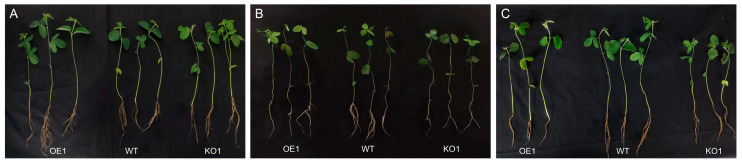
Phenotypes of different soybean materials under salt stress. (**A**) Under normal conditions; (**B**) 100 mmol^−1^ NaCl treatment; and (**C**) 200 mmol^−1^ NaCl treatment.

**Figure 11:**
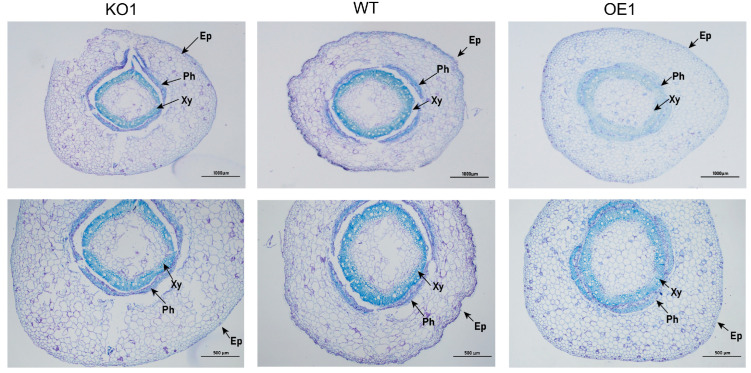
Transverse anatomical structure of soybean root under salt stress. Note: Ep, epidermal; Ph, phloem; Xy, xylem.

Soybean is a deep-rooted crop with wide and deep roots. With increasing salt concentration, the growth of different soybean materials was affected, showing varying degrees of yellowing, wilting, and other phenomena. The total root length and dry weight in over-expressing materials were significantly higher than those of the receptor control, while those of gene-edited materials were significantly lower than those of the receptor control (Table 2). The experimental results indicated that the growth of over-expressing materials was better than that of control and gene-edited materials (Figure 10). Next, the effects of salt stress on the soybean root microstructure were studied. As shown in Figure 11, after 15 days of 200 mmol/L NaCl treatment, the root anatomical structure results of the gene-edited strain and the recipient control indicated that the root epidermis of both strains showed internal and external depression, with some cells even breaking due to dehydration. The central cells were also damaged, while the root anatomy of the over-expressed strains remained relatively intact, the cells were arranged more neatly, and the central cells did not change significantly. In addition, there was no significant change in the root epidermis of gene-edited plants under salt stress when treated with 200 mmol/L NaCl, when compared with the control group. The xylem cells decreased significantly and the phloem cells became thinner. The xylem and phloem of over-expressing strains were significantly thicker than those of control plants. In conclusion, the strain with better salt tolerance may gain resistance to salt stress by reducing the thickness of the root epidermis and cortex and increasing the xylem and phloem thickness.

## 3. Discussion

The NDR1/HIN1-like (NHL) gene family has high homology with the non-specific resistance *NDR1* gene [23] and the tobacco Harpin-induced *HIN1* gene [24]. To date, many studies have shown that this gene family is related to plant defense [25,26,27]. In this study, the soybean *GmNHL1* gene was selected for cloning and stress resistance function analysis. The cis-acting elements of the promoter of the target gene were analyzed, and G-box, ABRE, and several elements related to stress were found. In previous studies, it has been shown that *CsbZIP50* combined with the G-box motif (TACGTG) or ABRE motif (ACGTG) in the promoter of *CsRD29A* can enhance the drought stress tolerance of cucumber by regulating stress response gene expression and ROS levels [28]. Therefore, we speculate that G-box and ABRE in the *GmNHL1* promoter may be regulated by alkaline leucine zips (bZIP) transcription factors in response to stress.

Many studies have verified the functions of genes through assessing phenotypic and physiological changes in transgenic strains. For example, it has been reported that over-expression of the soybean *GmLEA2-1* gene in transgenic *Arabidopsis thaliana* confers tolerance to salt stress. The growth and development performance of transgenic *Arabidopsis thaliana* were higher than that of wild *Arabidopsis thaliana* under salt stress. These results suggest that the *GmLEA2-1* protein plays an important role in improving the salt tolerance of plants [29]. PagWOX11/12a directly binds to the promoter region of PagCYP736A12 and regulates PagCYP736A12 expression. Over-expression of PagWOX11/12a in poplar enhanced salt tolerance by promoting growth-related biomass [30]. Plant water absorption is the process of water transport through the root epidermis and phloem layer of roots to the xylem ducts in the middle column. Therefore, changes in root anatomy are closely related to water absorption and transport in plants under stress [31].

Salt stress induces the generation of reactive oxygen species (ROS), and the excessive buildup of ROS can be detrimental to plant cell integrity. Consequently, it becomes essential to eliminate surplus ROS radicals and superoxide ions by stimulating the body’s enzymatic antioxidant system. Previous studies have reported that silencing of the PPR gene PPS1 in rice showed increased sensitivity to salt and ABA stress and led to significant ROS accumulation [32]. Seedling transfer of the MsMYB2L gene enhanced the synthesis of various osmoregulatory substances, such as proline and soluble sugar, and reduced lipid peroxidation, making this a potential candidate gene for manipulating salinity and drought tolerance in alfalfa [33]. In the present study, after salt stress, the chlorophyll content, proline content, and peroxidase activity of over-expressed *GmNHL1* transgenic lines were significantly higher than those in recipient control plants; meanwhile, the superoxide anion content and malondialdehyde content were significantly reduced. Therefore, we determined that *GmNHL1* increases the plant’s sensitivity to salt by reducing the accumulation of reactive oxygen species, thereby reducing the plant’s salt tolerance.

In this study, we mainly studied the function of the *GmNHL1* gene. Mutant plants of different genotypes were obtained through genetic transformation of gene knockout expression and over-expression vectors. The phenotype, physiology, biochemistry, and molecular experiments considering over-expressed *GmNHL1* transgenic soybeans under salt stress demonstrated that over-expression of *GmNHL1* increased the chlorophyll content, proline content, and peroxidase activity, while decreasing the contents of MDA and superoxide anions, thus improving the tolerance of the plants to salt stress. Phenotypic observations of the recipient soybean JN74, over-expressed soybean material, and gene-edited soybean material under salt stress indicated that the gene-edited line was superior to the recipient line and the recipient line was superior to the over-expressed line. The over-expressed lines showed better growth and development ability under salt stress, while the gene-edited lines showed earlier wilting and yellowing under salt stress. This experiment was consistent with previous research results, indicating that the soybean *GmNHL1* gene confers a certain resistance to abiotic stresses, thus providing a theoretical basis for breeding new soybean varieties with high yield.

## 4. Materials and Methods

### 4.1. Test Materials

The plant acceptor material was soybean variety Jinong 74. The strains and plasmids included *Agrobacterium tumefaciens* strain EHA105, and *Escherichia coli* strain DH5α, with pCAM-BIA3301 and pCAMBIA1302-GFP plant expression vectors provided by the Biotechnology Center of Jilin Agricultural University.

### 4.2. Cloning and Subcellular Localization of Genes

The NCBI database (https://www.ncbi.nlm.nih.gov/, accessed on 27 December 2022) was used to download the CDS *GmNHL1* gene sequence by RT-PCR reverse transcriptase gene clone. Blast was used to obtain the homologous gene sequences of *GmNHL1*. The MEGA11 software was used for multiple sequence comparisons to construct the phylogenetic evolutionary tree. The Conserved Domains Database (CDD) was used to analyze the conserved domains of candidate genes. In addition, according to the procedure outlined by Karikari et al. [34], the cis-acting elements of the promoter region of *GmNHL1* gene were further analyzed, and the cis-acting elements of the promoter region were visualized using TBtools.

In the sub-cellular localization test, *Agrobacterium* transformed into plasmid pCAMBIA1302 unloaded, and pCAMBIA1302-GmNHL1 was expanded and suspended referring to the instantaneous transformation method of tobacco [35]. The OD600 of the bacterial solution was adjusted to 0.6–0.8, and it was left for one hour at room temperature. The bacterial solution was injected into the leaves of 6–8-week-old tobacco plants and cultured overnight in the dark. After normal culture for three days, the fluorescence was observed using a laser confocal microscope (Nikon C2-ER, Tokyo, Japan).

In the artificial cultivation room, soybean variety Jinong 74 was planted. Five seeds were sown in each cup, and after germination, three seedlings were retained in each cup. When the soybeans reached the V1 growth stage with robust growth, they were subjected to stress treatments using 10% PEG-6000, 100 mmol/L NaCl, and maintained at 4 °C for varying durations. After different periods of stress treatment, soybean plant roots, stems, and leaves were collected (in triplicate experiments). Total RNA was extracted from the above-mentioned plant parts for fluorescence quantitative PCR reactions.

### 4.3. Construction of Plant Expression Vector

The plant over-expression vector was constructed using seamless cloning technology. The restriction endonucleases BglII and BstEII were used as the restriction sites to linearize the pCAMBIA3301 empty plasmid and the restriction endonucleases BglII and Nco I were used to linearize the pCAMBIA1302-GFP empty vector. The target gene *GmNHL1* fragment was constructed into the corresponding expression vector.

The CRISPR/Cas9 carrier was used to build the first use of the CRISPR -p website (http://crispr.hzau.edu.cn/CRISPR2/; accessed on 10 September 2022) for the purpose of designing the gene *GmNHL1* gRNA. The target (Table S1) mainly consisted of 20 bases, followed by NGG (N is any base), with three bases comprising the PAM region (Table S1). The constructed vector was a DNA plasmid with U6 as the promoter and B-GK041 (Cas/gRNA) as a carrier. Then, the CRISPR/Cas carrier construction kit provided by Baige Biotechnology Company was used to construct the CRISPR/Cas9 carrier, referring to the method of Wu et al. [19].

### 4.4. Genetic Transformation and Detection of Positive Plants

The *Agrobacterium tumefaciens*-mediated soybean cotyledon node method [36] was followed for genetic transformation. The soybean seeds were sterilized in a disinfection cabinet with 25 mL NaClO and 5 mL of concentrated HCl. After treatment, they were placed on prepared medium for germination for 3 days, pre-cultured for 3 days, and then subjected to *Agrobacterium* infection for 5 days. After about 15 days, the first screening was performed, followed by treatment with a herbicide at 1.2 mg·mL^−1^. Re-screening was conducted after 15 days, and the culture was resumed for 7 days. Finally, the screened callus was transferred to the elongation medium for 21 days, then transferred to rooting medium for culturing until the roots were robust and developed and the seedlings had developed well. Soybean gene-edited offspring were obtained through transplanting to indoor addition.

The PCR detection of positive plant-extracted DNA from young and tender parts of soybean (i.e., from the first group of triple compound leaves at V1 stage) was conducted using the new plant genome DNA extraction kit of Conweishi Company for DNA extraction. The Premier 5.0 software was used to label BAR-specific primers and Cas9 primers (Table S1) for screening.

Next, Southern blot hybridization of over-expressed *GmNHL1* gene strains was carried out. Referring to the method of Li et al. [37], CTAB was used to extract a large number of T2 generation PCR-transformed plants positive for genomic DNA detection. The Bar gene was used to prepare probes, the *GmNHL1* gene expression vector plasmid was used as the positive control, and the receptor CK was used as the negative control. The integration of the exogenous marker gene Bar in T2 generation soybean lines was detected.

### 4.5. Germination and Seedling Test

The germination test was conducted according to the methods of Zhou [38] and Zhang et al. [39]. First, 50 intact soybean seeds from each recipient soybean variety JN74, the over-expressed soybean material, and the gene-edited soybean material were selected, which were soaked in 75% alcohol and 5% NaCIO for 120 s, washed three times with distilled water, and then germinated in Petri dishes. NaCl solutions with different mass concentrations were set to 0 mmol/L, 100 mmol/L, and 200 mmol/L NaCl. Each concentration was added (at 20 mL) for the simulated salt stress treatments, and the control group was provided with the same amount of distilled water. The seeds were germinated in an incubator in an artificial climate (constant temperature of 25 °C, relative humidity of 70%). The germination condition was that the radicle broke through the seed coat by 1 mm, and the germ was half the length of the seed. The number of seeds that had germinated was recorded regularly every day, and GE and GR of each variety were calculated on the third and seventh days of cultivation. Ten germinated seeds were randomly selected for each replicate, and the root length and seedling height were measured with Vernier calipers. The germination rate (GR), germination potential (GE), germination index (GI), and vitality index (VI) of different soybean materials were determined according to the number of germinated seeds, using the following formulae:

GE = number of germinated seeds in 3 days/number of seeds tested,(1)

GI = ∑ (Gt/Dt),(2)

GR = number of germinated seeds in 7 days/number of seeds tested,(3)

VI = S × GI (GI: germination index, S: seedling growth),(4)

where Gt refers to the number of germinated seeds on day t and Dt denotes the corresponding germination day.

### 4.6. Determination of O_2_^−^ Content and Physiological and Biochemical Indexes in Soybean Plants

Soybean seeds of the recipient soybean variety JN74, over-expressed soybean material, and gene-edited soybean material were placed, seeded, and cultured in an artificial culture chamber (25 °C, 16 h of light/8 h of darkness). Then, physiological and biochemical data were measured under normal and salt stress conditions for 15 days.

Nitrotetrazolium chloride blue (NBT) histochemical staining was used to identify the ROS content in vivo, with specific reference to Fryer’s method [40]. The O_2_^−^ content in plants was calculated using the acetone method [41]. The malondialdehyde conditions were determined using the thiobarbituric acid method. The chlorophyll and proline contents were determined according to Nielsen’s method [42]. The activities of the SOD and POD antioxidant enzymes were determined by referring to the method of Wang [43].

### 4.7. Real-Time Quantitative PCR (RT-qPCR)

To detect the relative expression of target genes in different plant materials. RNAiso Plus (Takara Bio, Kyoto, Japan) was used to extract total RNA from the root, stem, and leaves of soybeans in the V1 period that tested positive using T2-generation PCR. These were then reverse-transcribed into cDNA using an All-in-One™ Firs t-Strand cDNA Synthesis Kit (GeneCopoeia Inc., Rockville, MD, USA), diluted by a factor of 5. The reaction procedure was 95 °C for 30 s, 95 °C for 10 s, and 60 °C for 30 s, for a total of 40 cycles. The soybean β-actin gene (GenBank entry number: NM_001252731.2) was selected as the internal reference gene, and the primer Quest Tool was designed online (https://sg.IDTDNA.com/pages/tools; accessed on 10 September 2022; see Table S1). To evaluate the reproducibility and stability of the experiment, we selected three biological replicates. The analysis was performed using Mx3000P fluorescent quantitative PCR (Agilent Technologies, Lexington, MA, USA). The 2^−ΔΔCt^ formula was used to calculate the expression. Histograms were plotted using the GraphPad Prism 9.5.0 software (https://www.graphpad-prism.cn/) (accessed on 3 January 2023).

### 4.8. Phenotypic Analysis of Soybean under Different Degrees of Salt Stress

A root scanner was used to scan the total root length. The soybean samples were put into an aluminum box with known weight, then put in a 105 °C oven for 15 min after drying, and then set to 80 °C until a constant weight was reached. Then, the dry weight was measured. The root structure of the soybean plants was assessed by slicing and using paraffin wax, slightly improved by referring to Yu’s [44] method. On the 15th day after salt stress treatment at 200 mmol/L NaCl, soybean roots were removed from the soil matrix as completely as possible, the root matrix was washed with water, and water was sucked up with absorbent paper. The root segment (cross-section) was cut from the middle part of the main root. The plant tissue material was immediately fixed in a formalin fixing solution (FAA) for more than 48 h. Using tert-butanol gradient dehydration, sections with a thickness of 16 μm were obtained using a microtome (LeicarRM2245, Vizsla, Germany). The paraffin sections were stained with toluidine blue, then observed and photographed using an orthofluorescence microscope (Nikon ECLIPSE, Tokyo, Japan) after staining.

### 4.9. Statistical Analysis

All experiments in this study were independently repeated three times. The SPSS 23.0 software was used for statistical analysis of experimental measurement data, and one-way ANOVA was conducted to verify the variability of results between different treatments. In statistics, results with significance are labeled as * *p* < 0.05, while results with a significance level less than 0.01 are labeled as ** *p* < 0.01.

## 5. Conclusions

In this study, we mainly studied the function of the *GmNHL1* gene. Mutant plants of different genotypes were obtained through genetic transformation using gene knockout expression and over-expression vectors. Phenotype, physiology, biochemistry, and molecular analyses of over-expressed *GmNHL1* transgenic soybeans under salt stress demonstrated that over-expression of *GmNHL1* increased the chlorophyll content, proline content, and peroxidase activity while decreasing the contents of MDA and superoxide anions, thus improving the salt stress tolerance of the plants. Phenotypic observation of the three different genotypes under salt stress indicated that the gene-edited lines were inferior to the receptor lines, while the receptor lines were inferior to the overexpressed lines. The over-expressed lines showed better growth and development ability under salt stress, while the gene-edited lines showed earlier wilting and yellowing under salt stress. These results are consistent with previous research, indicating that the soybean *GmNHL1* gene can confer a certain degree of abiotic stress resistance, thus providing a theoretical basis for breeding new soybean varieties with high yield.

## Figures and Tables

**Table 1:** Germination indices of soybean under salt stress in different plant materials.

**Determination of Indicators**	**Normal**				
	**JN74**	**OE1**	**OE2**	**KO1**	**KO2**
GR (%)	98.45 ± 1.67	99.12 ± 2.16 *	98.33 ± 1.67 *	98.33 ± 1.67	98.33 ± 1.67
GI	9.21 ± 0.03	9.57 ± 0.10 *	9.34 ± 0.27 *	9.51 ± 0.18	9.21 ± 0.14
GE (%)	78.45 ± 0.12	77.61 ± 0.16 *	77.82 ± 0.28 *	78.56 ± 1.01	77.27 ± 1.12
VI	6.47 ± 0.12	7.39 ± 0.12 *	8.71 ± 0.21 *	7.01 ± 0.13	7.12 ± 0.14 *
**Determination of Indicators**	**100 mmol L^−1^ NaCl**				
	**JN74**	**OE1**	**OE2**	**KO1**	**KO2**
GR (%)	98.44 ± 1.14	98.05 ± 1.70 **	98.69 ± 2.89 **	96.67 ± 1.67 **	97.33 ± 2.67 **
GI	7.73 ± 0.17	8.61 ± 0.12 **	9.08 ± 0.18 **	6.71 ± 0.21 **	7.03 ± 0.18 **
GE (%)	65.05 ± 0.15	68.15 ± 0.16 **	66.16 ± 0.95 **	63.54 ± 0.85 **	62.96 ± 0.75 **
VI	5.38 ± 0.14	6.56 ± 0.13 **	6.81 ± 0.12 **	4.55 ± 0.14 **	3.83 ± 0.12 **
**Determination of Indicators**	**200 mmol L^−1^ NaCl**				
	**JN74**	**OE1**	**OE2**	**KO1**	**KO2**
GR (%)	68.33 ± 2.01	86.76 ± 2.26 **	90.00 ± 1.77 **	69.67 ± 1.41 **	61.67 ± 1.28 **
GI	4.08 ± 0.25	5.71 ± 0.18 **	5.19 ± 0.23 **	3.95 ± 0.17 **	3.47 ± 0.22 **
GE (%)	47.45 ± 0.23	59.38 ± 0.41 **	54.36 ± 0.36 **	46.84 ± 0.46 **	47.35 ± 0.67 **
VI	1.83 ± 0.12	2.24 ± 0.12 **	2.32 ± 0.11 **	1.71 ± 0.15 **	1.12 ± 0.11 **

Note: GR: germination rate; GE: germination potential; GI: germination index; VI: vitality index. * *p* < 0.05; ** *p* < 0.01.

**Table 2:** Determination of germination finger of soybean under salt stress for different plant materials.

	**Normal**				
	**JN74**	**OE1**	**OE2**	**KO1**	**KO2**
The dry weight (g)	0.32 ± 0.15	0.35 ± 0.23	0.37 ± 0.26	0.33 ± 2.31	0.31 ± 0.76
Total root length (cm)	129 ± 1.21	172.41 ± 0.77	158.42 ± 0.91	102.51 ± 1.73	99.71 ± 2.41
	**100 mmol L^−1^ NaCl**				
	**JN74**	**OE1**	**OE2**	**KO1**	**KO2**
The dry weight (g)	0.39 ± 0.17	0.45 ± 0.25*	0.47 ± 1.31 *	0.35 ± 1.74 *	0.34 ± 2.14 *
Total root length (cm)	131.65 ± 0.26	175.53 ± 2.61**	160.13 ± 0.89 **	105.74 ± 2.97 **	100.76 ± 3.45 **
	**200 mmol L^−1^ NaCl**				
	**JN74**	**OE1**	**OE2**	**KO1**	**KO2**
The dry weight (g)	0.43 ± 2.01	0.56 ± 2.26 *	0.61 ± 1.77 *	0.39 ± 1.41 *	0.38 ± 1.28 *
Total root length (cm)	135.61 ± 1.11 **	178.45 ± 0.76 **	166.41 ± 2.35 **	107.41 ± 0.87 **	103.28 ± 1.01 **

* *p* < 0.05; ** *p* < 0.01.

## Data Availability

Data are contained within the article and Supplementary Materials.

## Supplementary Materials

The following supporting information can be downloaded at: https://www.mdpi.com/article/10.3390/plants12223869/s1, Figure S1: Bioinformatics analysis of *GmNHL1* gene. (A) Phylogenetic tree of *NHL* family genes in rice, corn and soybean; (B) Analysis of cis-acting elements in promoter region; (C) Conserved domain of *GmNHL1* gene analyses; (D) Multiple sequence alignment of *NHL* family genes in rice, corn and soybean. Table S1: PCR primers were detected.
