# Supplementary material for: Cloning of the Soybean GmNHL1 Gene and Functional Analysis under Salt Stress
Source: Plants (Basel). 2023 Nov 16;12(22):3869. doi: 10.3390/plants12223869 (PMC10675494; doi:10.3390/plants12223869)
Supplement: Supplementary file 1 [file plants-12-03869-s001.zip › plants-2695666-supplementary.pdf]

Table S1.PCR primers were detected

**Name**	**Sequence（5′ -3′）**
Gm NHL1-S	TTCTCAGCCAAACAATCC
Gm NHL1-AS	TAGGGCTGACGGTGAAGAA
Gm NHL1-S	agaacacgggggactcttgaTTCTCAGCCAAACAATCC
GmNHL1-AS	cgatcggggaaattcgagctTAGGGCTGACGGTGAAGAA
1302AS	ATGGTACAGAAAAAGGTTCTTTC
1302S	CACTTTTTTGGTCATTCATACGCTC
Bar-S	TCAAATCTCGGTGACGGGC
Bar-AS	ATGAGCCCAGAACGACGCC
35S-S	TAGAGGACCTAACAGAAC
35S-AS	CCGTGTTCTCTCCAAATG
NOS-S	GAATCCTGTTGCCGCTCTTG
NOS-AS	TTATCCTAGTTTGCGCGCTA
Cas9-S	CCCAAGAGGAACAGCGATAAG
Cas9-AS	GTCGATGGTGGTGTCAAAGT
Actin2-S	CTGAGGTTCTATTCCAGCCATCC
Actin2-AS	CCACCACTGAGGACAACATTACC
QGmNHL1-S	CTCACCCAAGACGACCTCAAG
QGmNHL1-AS	ATGCCGGACTCGACGTACTC
**Target Sequence+PAM**	**SG Sequence**
GTAGACCGTGACGTCTTGGAGG	GTGAACGGAATTGAACGG
AGATTACGTAGATTTCGAGGCGG	GTGTTTGGAACCCTTGAGAG

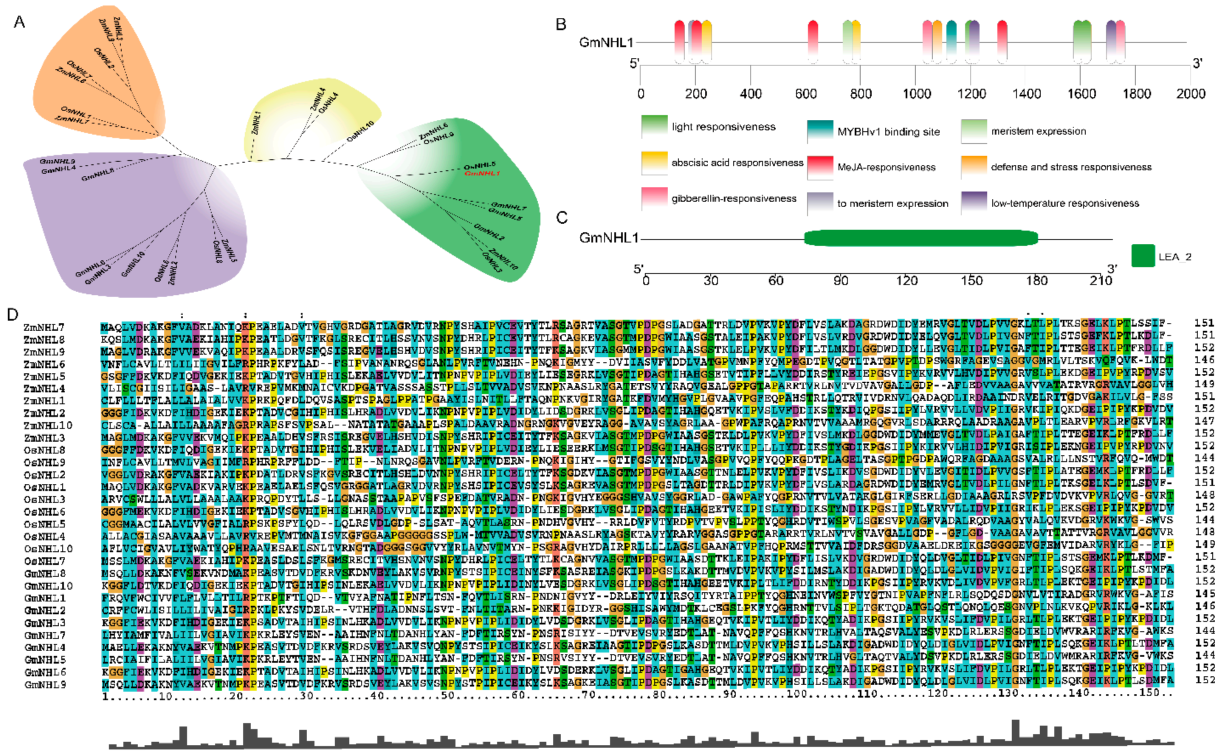

Figure S1: Bioinformatics analysis of *GmNHL1* gene. (A) Phylogenetic tree of *NHL* family genes in rice, corn and soybean; (B) Analysis of cis-acting elements in promoter region; (C) Conserved domain of *GmNHL1* gene analyses; (D) Multiple sequence alignment of *NHL* family genes in rice, corn and soybean
